# Supplementary figures and images for: Genome-Wide Identification of Peanut B-Boxs and Functional Characterization of AhBBX6 in Salt and Drought Stresses
Source: Plants (Basel). 2024 Mar 26;13(7):955. doi: 10.3390/plants13070955 (PMC11013918; doi:10.3390/plants13070955)

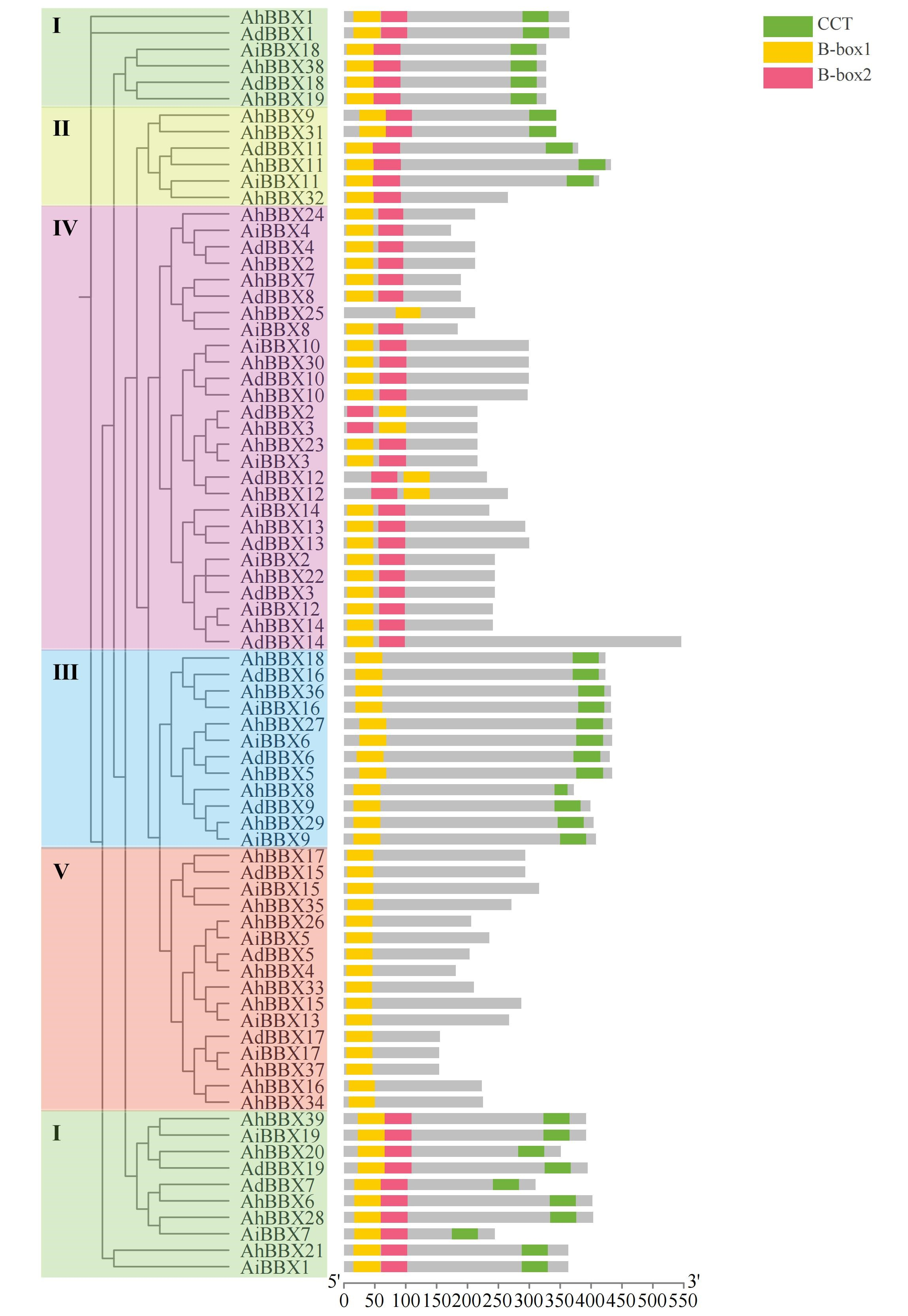

Supplement: Supplementary file 1 [file plants-13-00955-s001.zip › Fig S1.tiff]

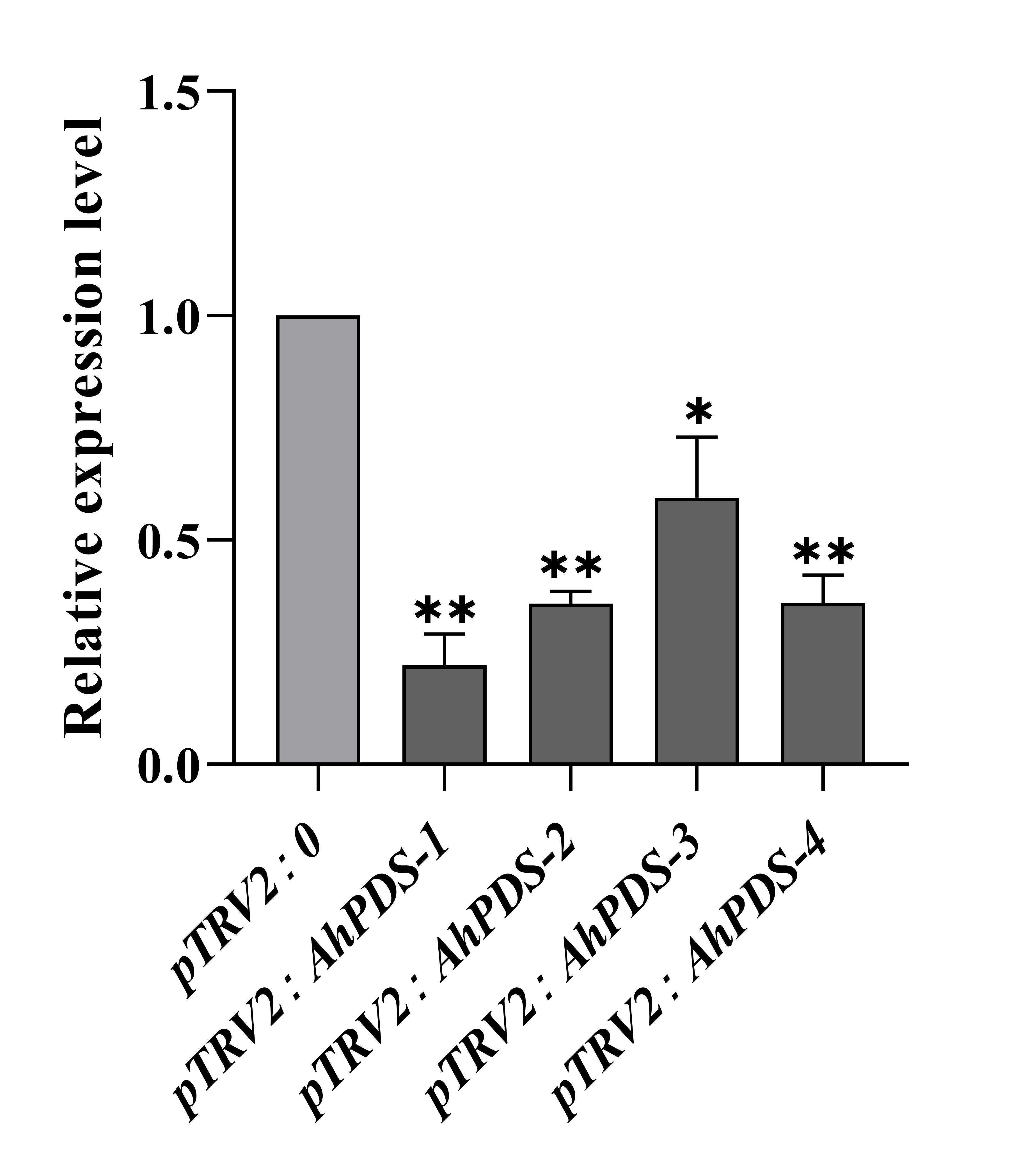

Supplement: Supplementary file 1 [file plants-13-00955-s001.zip › Fig S2.tiff]

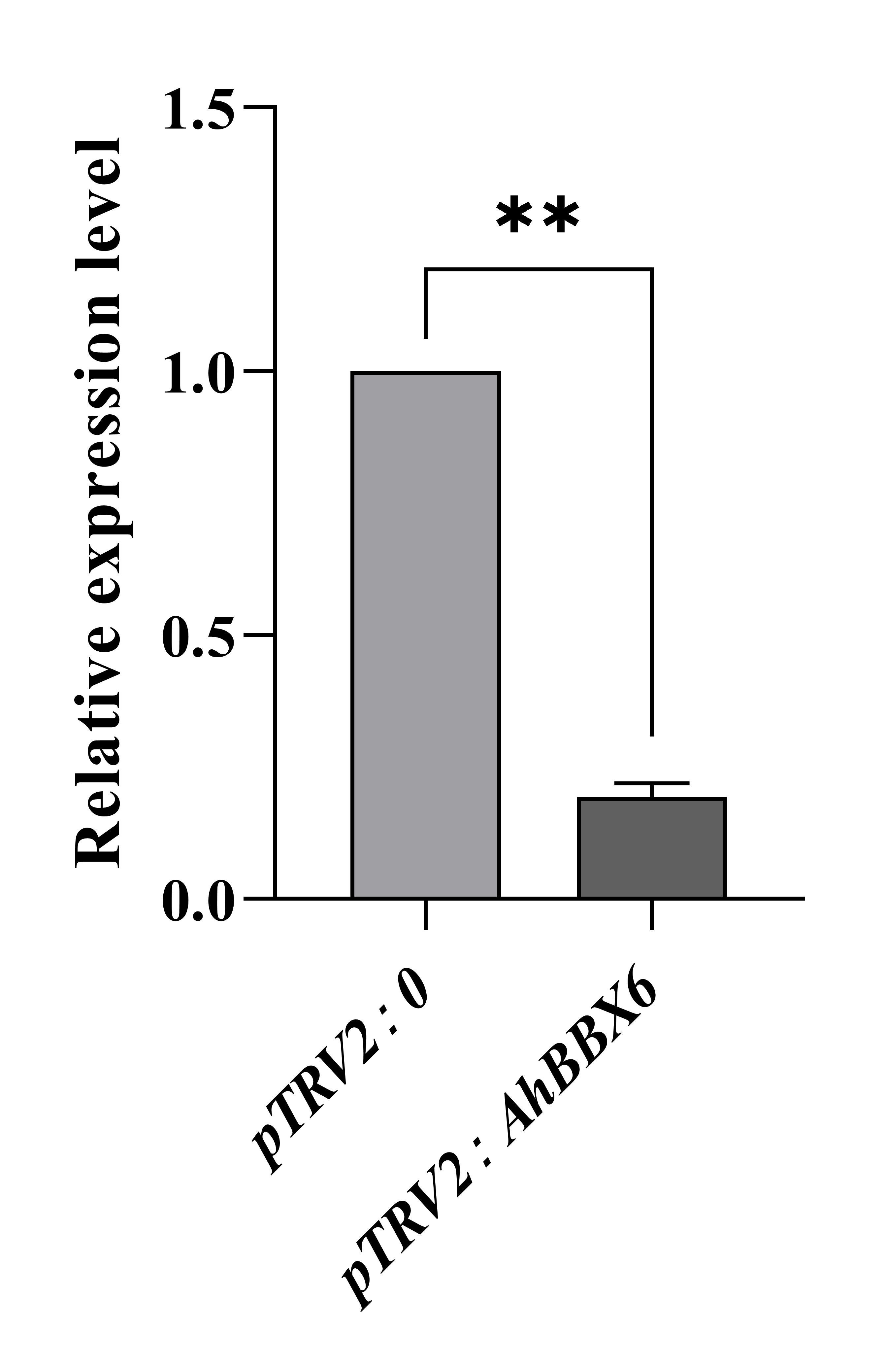

Supplement: Supplementary file 1 [file plants-13-00955-s001.zip › Fig S3.tiff]

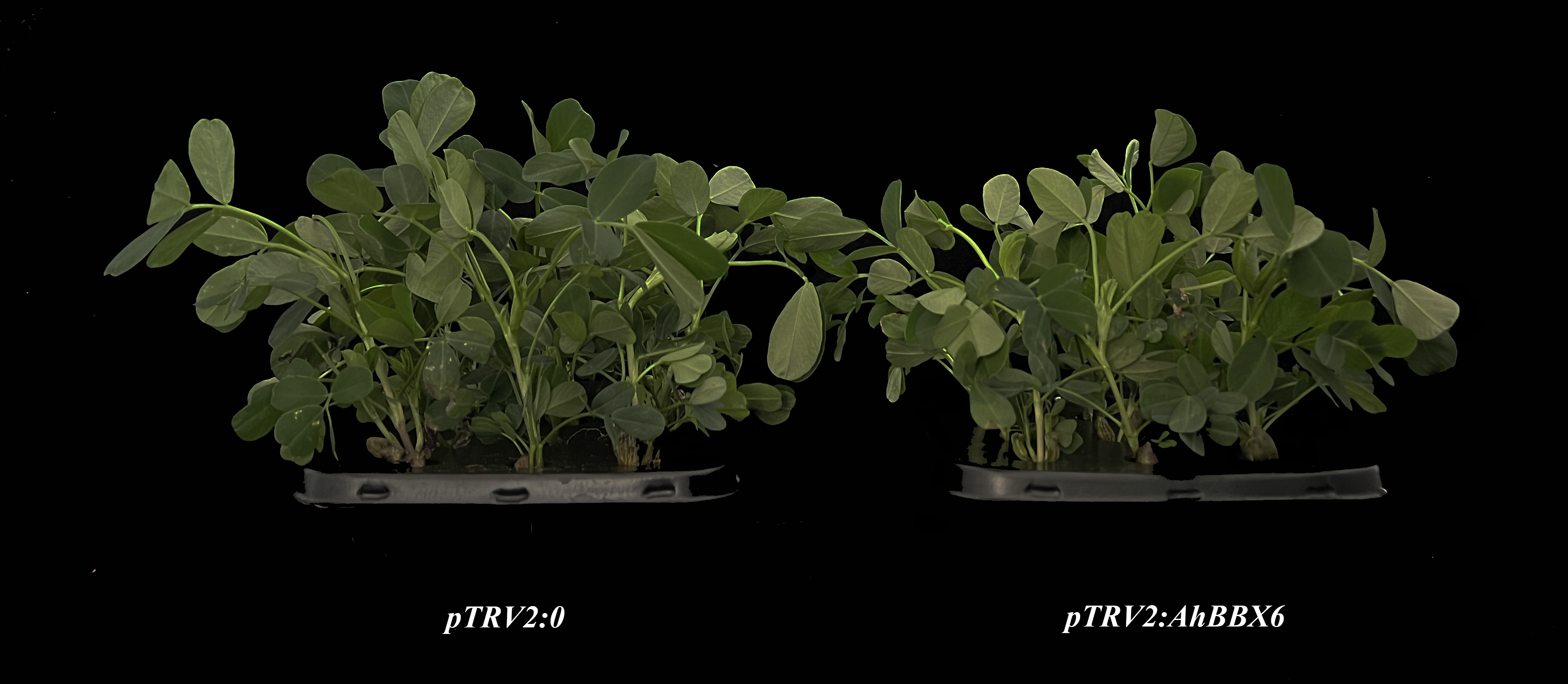

Supplement: Supplementary file 1 [file plants-13-00955-s001.zip › Fig S4.tif]
